# Genome-Wide Target Analyses of Otx2 Homeoprotein in Postnatal Cortex

**DOI:** 10.3389/fnins.2017.00307

**Published:** 2017-05-31

**Authors:** Akiko Sakai, Ryuichiro Nakato, Yiwei Ling, Xubin Hou, Norikazu Hara, Tomoya Iijima, Yuchio Yanagawa, Ryozo Kuwano, Shujiro Okuda, Katsuhiko Shirahige, Sayaka Sugiyama

**Affiliations:** ^1^Laboratory of Neuronal Development, Graduate School of Medical and Dental Sciences, Niigata UniversityNiigata, Japan; ^2^Research Center for Epigenetic Disease, Institute of Molecular and Cellular Biosciences, University of TokyoTokyo, Japan; ^3^Bioinformatics Laboratory, Graduate School of Medical and Dental Sciences, Niigata UniversityNiigata, Japan; ^4^Department of Molecular Genetics, Center for Bioresources, Brain Research Institute, Niigata UniversityNiigata, Japan; ^5^Department of Genetic and Behavioral Neuroscience, Graduate School of Medicine, Gunma UniversityGunma, Japan

**Keywords:** Otx2, parvalbumin, ChIP-seq, critical period, neurodevelopmental disorder

## Abstract

Juvenile brain has a unique time window, or critical period, in which neuronal circuits are remodeled by experience. Mounting evidence indicates the importance of neuronal circuit rewiring in various neurodevelopmental disorders of human cognition. We previously showed that Otx2 homeoprotein, essential for brain formation, is recaptured during postnatal maturation of parvalbumin-positive interneurons (PV cells) to activate the critical period in mouse visual cortex. Cortical Otx2 is the only interneuron-enriched transcription factor known to regulate the critical period, but its downstream targets remain unknown. Here, we used ChIP-seq (chromatin immunoprecipitation sequencing) to identify genome-wide binding sites of Otx2 in juvenile mouse cortex, and interneuron-specific RNA-seq to explore the Otx2-dependent transcriptome. Otx2-bound genes were associated with human diseases such as schizophrenia as well as critical periods. Of these genes, expression of neuronal factors involved in transcription, signal transduction and mitochondrial function was moderately and broadly affected in Otx2-deficient interneurons. In contrast to reported binding sites in the embryo, genes encoding potassium ion transporters such as K_V_3.1 had juvenile cortex-specific binding sites, suggesting that Otx2 is involved in regulating fast-spiking properties during PV cell maturation. Moreover, transcripts of oxidative resistance-1 (*Oxr1*), whose promoter has Otx2 binding sites, were markedly downregulated in Otx2-deficient interneurons. Therefore, an important role of Otx2 may be to protect the cells from the increased oxidative stress in fast-spiking PV cells. Our results suggest that coordinated expression of Otx2 targets promotes PV cell maturation and maintains its function in neuronal plasticity and disease.

## Introduction

Animal behavior often reflects brain function refined by experience in early postnatal life (Hensch, 2004). Recent evidence suggests that aberrant remodeling of neuronal networks underlies many forms of human neurodevelopmental disorders (Leblanc and Fagiolini, 2011). Neuronal circuits are first established by genetic programs and intrinsic activity and are then sharpened by individual experience during a distinct time window called the critical period. Elevated plasticity during critical periods is essential for rewiring circuits capable of relevant cognition that are sustained into adulthood (Takesian and Hensch, 2013). Specific gene regulation, which depends on environmental cues and neuronal activity, is required for such remodeling of functional circuits. Gene expression patterns can be defined by batteries of transcription factors and by the chromatin environment that is interrelated with transcription factor binding. Accumulating evidence suggests that deficiencies in transcription factors and chromatin modification factors affects the development of neuronal networks, leading to neurodevelopmental disorders such as schizophrenia and autism spectrum disorder (Ronan et al., 2013; De Rubeis et al., 2014).

The homeodomain transcription factor Otx2 is critical for embryonic and postnatal development of the nervous system, with many experimental studies indicating its essential role in the eyes (Bovolenta et al., 1997; Nishida et al., 2003; Kim et al., 2015), forebrain (Acampora et al., 2001; Tian et al., 2002; Nakamura and Sugiyama, 2004), and pituitary (Matsuo et al., 1995; Mortensen et al., 2011), and in the specification of neuronal subtypes such as midbrain dopaminergic neurons (Puelles et al., 2004; Omodei et al., 2008) and thalamic GABAergic interneurons (Golding et al., 2014). In humans, *Otx2* mutations have been linked to dysfunctions of the brain and eyes (Béby and Lamonerie, 2013). At the chromatin level, Otx2 functions as an activator or repressor of transcription, depending on its interacting partners. For instance, during embryonic stem cell differentiation, Otx2 induces enhancer activation by recruiting p300 histone acetyltransferase (Yang et al., 2014). It also mediates transcription inhibition via binding to Groucho/TLE (Agoston and Schulte, 2009; Yasuoka et al., 2014).

We previously reported that Otx2 functions as an inducer of the critical period in mouse postnatal visual cortex (Sugiyama et al., 2008). Otx2 plays a key role in promoting the maturation of cortical GABAergic interneurons, which is especially important for triggering the critical period (Hensch et al., 1998). Interestingly, Otx2 is not expressed in posterior cortex but the protein is transported into cortical interneurons, mainly parvalbumin-positive (PV) cells, from visual pathways (Sugiyama et al., 2008) and the choroid plexus (Spatazza et al., 2013). Otx2 protein is preferentially internalized by binding with chondroitin sulfate proteoglycan (CSPG) in the perineuronal net (PNN) around PV cells (Beurdeley et al., 2012). Importantly, the incorporation and function of Otx2 in PV cells depend on visual experience, thus positioning Otx2 as a mediator of “experience” (not just spike activity) in maturating cortical circuits (Sugiyama et al., 2009). Several studies have investigated developmental and/or activity-dependent gene expression during critical periods (Ossipow et al., 2004; Majdan and Shatz, 2006; Tropea et al., 2006; Lyckman et al., 2008; Benoit et al., 2015), including transcriptome analysis of cortical fast-spiking interneurons during their maturation (Okaty et al., 2009). However, downstream targets of Otx2 in postnatal cortical interneurons are totally unknown.

Recent genome-wide target analyses have demonstrated the roles of Otx2 in cellular specification and function at different developmental stages and in various tissues, such as the embryonic ganglionic eminence (Hoch et al., 2015) and postnatal eyes (Housset et al., 2013; Samuel et al., 2014). In mature neural retina and retinal pigment epithelium (RPE), in which Otx2 has specialized functions respectively, the majority of Otx2 binding genomic sites and target genes do not overlap (Samuel et al., 2014). Thus, in postnatal cortex, Otx2 may also exert unique functions by targeting distinct genes. In the present study, by ChIP-seq (chromatin immunoprecipitation sequencing) and cell-type specific RNA-seq analyses, we investigated Otx2 target genes in juvenile cortical interneurons where Otx2 is, to date, the only known cell-type specific transcription factor for critical period plasticity. Our results suggest novel targets of Otx2 for its supporting role in the development and functional integrity of postnatal PV interneurons.

## Results

### Identification of Otx2-bound chromatin regions in juvenile cortex

**Figure 1:**
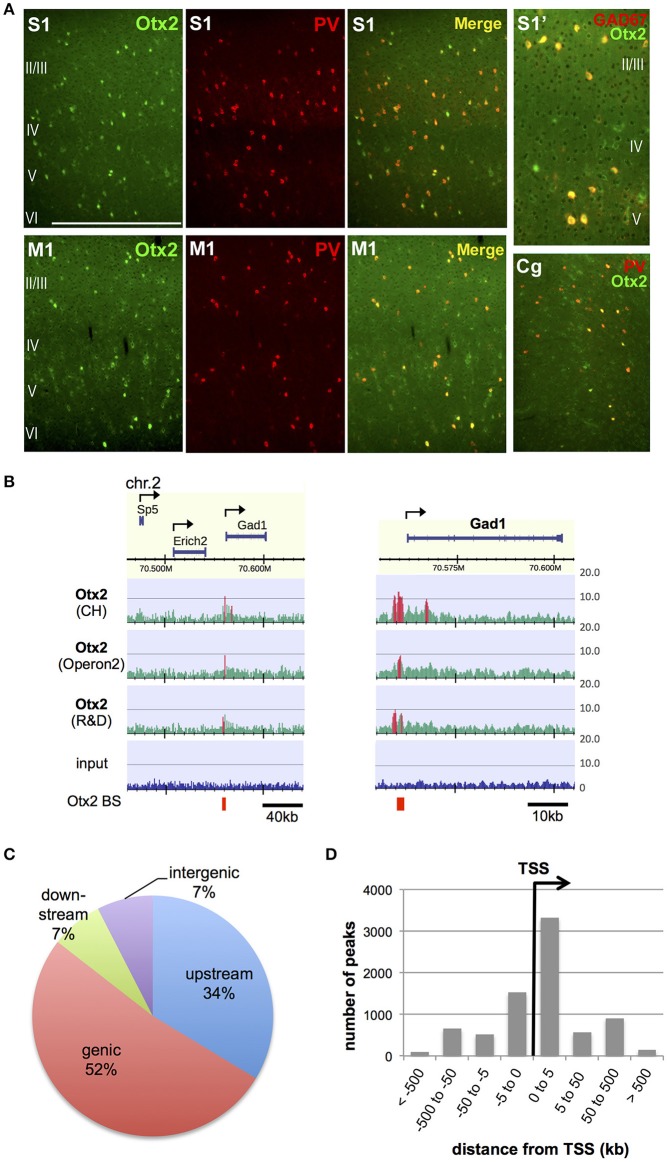
Identification of genomic Otx2 binding sites in postnatal cortices. **(A)** Otx2 localization in PV cells in the primary somatosensory (S1), motor (M1), and cingulate (Cg) areas at P28. Note that a few Otx2-positive cells do not have GAD67 signal in somatosensory cortex (S1′). The scale bar represents 500 μm for S1, M1, and Cg, and 250 μm for S1′. **(B)** Representative genomic maps of Otx2 binding sites (left) and magnified view of the *Gad1* genomic region with Otx2 binding sites (right). *Gad1* promoter possesses an Otx2 peak in all three ChIP-seq using different antibodies. Vertical axis shows read intensity normalized by total mapped read number. Peak regions identified by MACS are shown in red within and beneath the map. Arrows indicate TSS and direction of transcription. BS, binding site. **(C)** Distribution of genomic feature of Otx2 binding sites. Upstream and downstream indicate regions within −5 kb of the TSS and +5 kb from the transcription end site, respectively. **(D)** Distance distribution of Otx2 binding sites with respect to nearest TSS, analyzed using GREAT. Note that a peak may be assigned to more than one adjacent genes.

In order to identify Otx2 binding sites in the genome of postnatal mouse cortex, we attempted ChIP-seq analysis. Previous studies have indicated that cortical Otx2 proteins are provided by extracellular sources and transported mainly into PV cells throughout the cerebral cortex (Sugiyama et al., 2008; Spatazza et al., 2013). Consistent with these studies, Otx2 proteins in juvenile (P28) mice were enriched mostly in PV cells in the cingulate, primary motor and somatosensory areas as well as in the primary visual cortex (V1) (Figure 1A). Some Otx2 protein was distributed outside GAD67-positive interneurons, suggesting the existence of Otx2-positive pyramidal neurons (Figure 1A); however, we expected that the Otx2 ChIP signals would represent binding events mainly in interneurons. Because of the low abundance of cortical Otx2 and the similar populations of Otx2-positive cells across the cortex, ChIP-seq analysis was performed using a large part of the P28 cortex, in which neuronal circuits are more plastic during postnatal development than in adulthood. To increase reliability, we used three independent antibodies (see Section Materials and Methods), two of which (CH and Operon2) can be used in immunohistochemistry studies to detect nuclear Otx2 within cortical PV cells, and the other (R&D) having been used in previous ChIP-seq studies in different systems (Samuel et al., 2014; Hoch et al., 2015). The quality of each ChIP was confirmed by ChIP-qPCR analysis to detect enrichment of the Otx2 binding sites identified in our preliminary ChIP-seq trial (Figure S1).

In ChIP-seq analysis, peak calling in comparison to input was carried out using MACS software (Figure 1B, see Section Materials and Methods) and 13,596, 8,845, and 4,332 peaks were obtained for samples using CH, Operon2, and R&D antibodies, respectively. We focused on the peaks that overlapped between ChIP samples from at least two antibodies so that relatively weak but important peaks could be included while excluding false positives (Figure S2A). Under this consensus peak set, 5,490 peaks were identified as Otx2 binding sites in P28 cortex. We searched for peaks containing the consensus binding sequences of Otx2 and found that 1,273 (23.2%) possessed the major target sequence 5′-TAATCC-3′. In addition, two related motifs, 5′-TAAGCC-3′ and 5′-TAACCC-3′ (Chatelain et al., 2006), were found in 1,369 and 1,262 peaks, respectively. In total, 2,253 (41%) of our common peak set contained Otx2 consensus and/or related motifs, confirming the reliability of the data. As expected from Otx2 homeoprotein being a transcription factor, there were prominent peak signals at specific gene promoter regions over the mouse genomic map (Figure 1B). Analyses of peak positions relative to genes demonstrated that the majority of peaks were within genes (genic: 52%) and in the 5 kb region upstream (34%; Figure 1C). Notably, when peak locations were compared to transcription start sites (TSS), nearly half of the peaks were positioned within 5 kb downstream of a TSS, suggesting that most “genic” signals were located near TSS (Figure 1D). Examining the peaks both within gene and in the 5 kb upstream/downstream vicinity, a total of 5,199 genes were annotated to have Otx2 binding sites.

**Figure 2:**
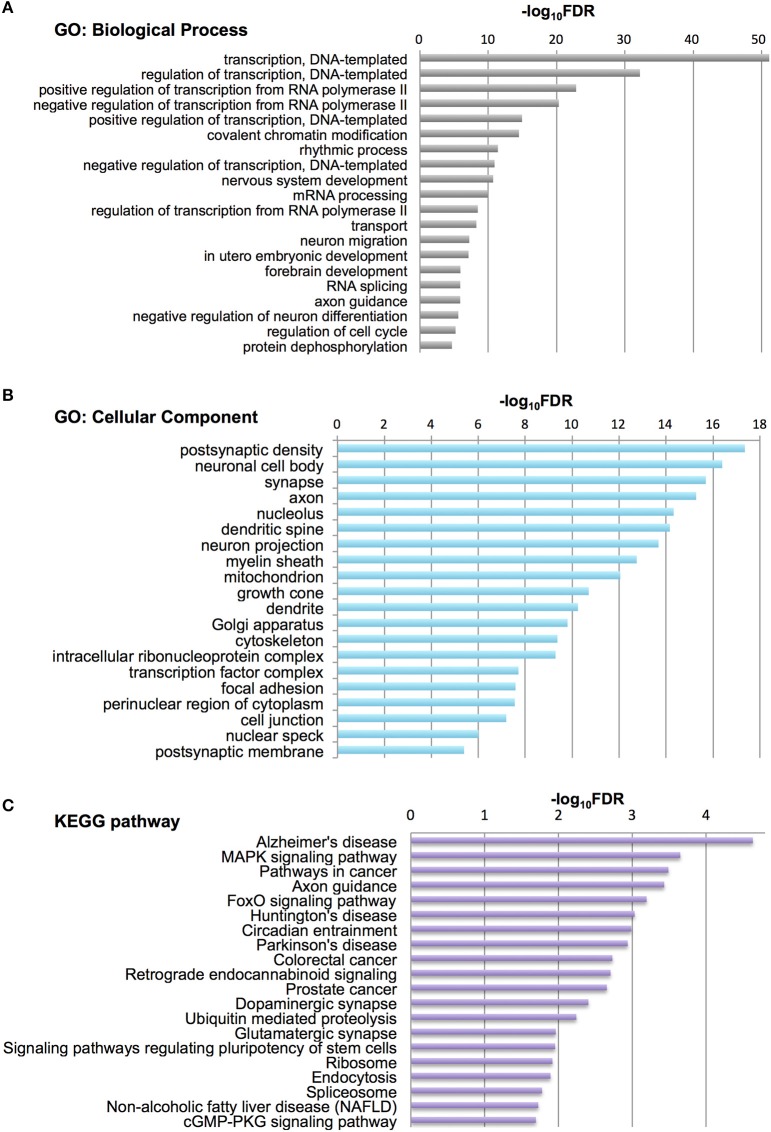
Category of genes with Otx2 binding sites. **(A,B)** Top 20 gene ontology (GO) terms in biological process **(A)** or cellular component **(B)** categories enriched in Otx2-bound genes. GO terms are ranked by false discovery rate (FDR). **(C)** Top 20 KEGG pathways enriched in Otx2-bound genes, ranked by FDR.

To characterize Otx2-bound genes, we investigated their ontology using DAVID (the Database for Annotation, Visualization and Integrated Discovery; Huang et al., 2009a,b). In the biological process category, the top six enriched terms were related to transcriptional regulation and chromatin modification, suggesting that Otx2 may regulate transcriptional cascades in postnatal cortex (Figure 2A). In addition, there were categories related to nervous system and forebrain development including migration and axon guidance, as well as particular functions such as rhythmic process and transport. Gene ontology (GO) of cellular component categories further confirmed enrichment of neuronal components such as axon, synapse, neuron projection, and myelin sheath (Figure 2B). Of note, the term “mitochondrion” was also enriched, probably reflecting the functional importance of energy metabolism in PV cells and the potential role of Otx2 in its regulation. This notion was strengthened by the fact that the enriched KEGG pathway contained a number of neurodegenerative diseases such as Alzheimer's, Huntington's, and Parkinson's diseases, whose common components are mitochondrial factors (Figure 2C). In addition, enrichment of the FoxO signaling pathway, implicated in the oxidative stress response, further suggested that Otx2 targets may involve factors for the maintenance of cellular integrity. Correspondingly, functional disease ontology analysis showed that genes related to “Alzheimer's disease” and “Schizophrenia” were enriched (Table 1), and that those related to schizophrenia (Hess et al., 2016; Wu et al., 2016b) indeed possessed Otx2-binding sites around the TSS (Table S1). Together, ChIP-seq data in P28 cortex revealed potential Otx2 targets that regulate transcription, neuronal development and mitochondrial function and are implicated in cellular integrity and disease.

**Table 1:** Enriched Disease Ontology (DO) terms for Otx2 binding sites.

**DO term**	**Number of genes**	**Bonferroni corrected *p*-value**
Alzheimer's disease	73	3.36E−22
Schizophrenia	54	5.34E−12
Bipolar disorder	34	6.38E−12
Brain tumor	49	8.64E−11
Down syndrome	31	1.17E−09
Epilepsy	24	3.41E−09
Congenital abnormality	44	3.53E−06
Amyotrophic lateral sclerosis	21	1.07E−05

*Terms related to cancer are excluded except for brain tumor*.

### Comparison of Otx2 binding sites with embryonic brain, neural retina, and RPE

Distinct genomic Otx2 binding sites have recently been reported in different tissues and developmental stages (Samuel et al., 2014; Yang et al., 2014; Hoch et al., 2015). It prompted us to compare Otx2 peaks with other systems to identify unique Otx2 binding sites in postnatal cortex. Raw data from previous studies were used for our peak calling to minimize analytical differences between laboratories. The same antibody (R&D) was also used for ChIP experiments in all previous studies and ours, to further reduce differences in experimental conditions between groups. We found that 191 peaks were shared among all four systems including P28 cortex, embryonic subpallium, neural retina and RPE (Figures S2B,C) despite the distinct functions and genomic targets of Otx2 depending on cellular context (Samuel et al., 2014). Conversely, P28 cortex also had unique Otx2 binding sites (2,683 peaks) compared to embryonic subpallium, neural retina and RPE.

**Figure 3:**
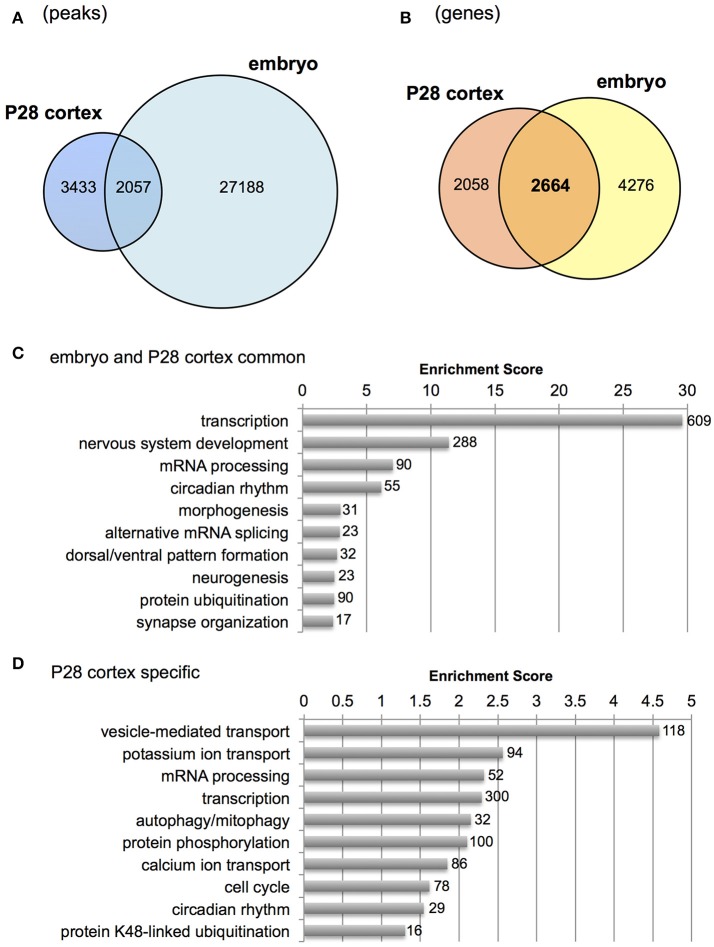
Comparison of Otx2 peaks with embryonic forebrain. **(A)** Venn diagram showing overlap of Otx2 peaks in P28 cortex and embryonic subpallium (embryo). **(B)** Venn diagram showing overlap of genes carrying Otx2 binding sites within ±5 kb of TSS in P28 cortex and embryonic subpallium (embryo). **(C,D)** Top 10 GO clusters enriched in Otx2-bound genes that are common to embryo and P28 cortex **(C)** or specific to P28 **(D)** in biological process categories. Each cluster is shown by a representative term. Number of all genes included in each cluster is shown.

Next, we examined genes bound by Otx2 in postnatal cortex but not in embryonic subpallium which includes progenitors of cortical interneurons at the ganglionic eminence. Because the published embryonic data contain only a few hundred common peaks among triplicate datasets (Hoch et al., 2015), we used “replicate-3 (replica3)” data of them, which produced a large number of peaks (29,533) in our peak calling, allowing us to find as many common peaks between embryo and P28 cortex as possible. As a result, over 2,000 overlapping peaks were detected between P28 cortex and embryo (Figure 3A). Next, we focused on genes carrying Otx2 binding sites in the 5 kb upstream and downstream from the TSS to include the peaks that did not overlap exactly between embryo and P28 cortex but existed in the promoter region of same gene. Comparison of P28 cortex (4,722 genes in total) with embryonic subpallium (6,940 genes in total) revealed that 2,664 genes appeared in both P28 and embryo, suggesting overlapping targets of Otx2 during development (Figure 3B). Common genes (2,664) or P28-specific genes (2,058) were classified based on GO terms of biological processes (Tables S2, S3) and subjected to gene clustering analysis (Figures 3C,D). We found that terms related to “development of nervous system and forebrain” appeared only in common genes (Figure 3C, Table S2). In fact, genes encoding transcription factors involved in the development of the forebrain (such as *Emx1/2, Otx1/2*, and *Pax6/7*) or interneurons (*Dlx2, Foxg1*, and *Etv1*) were bound by Otx2 in embryonic and P28 cortex (Figure S2D and data not shown). These results suggest that Otx2 may directly regulate these genes by binding to their promoter both in embryonic progenitors and in postnatal interneurons. Categories such as “transcription” and “mRNA processing” enriched in both common and P28-specific genes, indicating that these functions were maintained in embryonic and postnatal development via common genes (Figure 3C) and that postnatally, P28-specific genes were added in the same categories (Figure 3D). In contrast, terms implicated in “transport,” “protein catabolic process,” “signal transduction,” and “phosphorylation” were unique to P28-specific Otx2 targets (Figure 3D, Table S3).

Gene clusters containing “vesicle-mediated transport,” “potassium/calcium ion transport,” and “autophagy/mitophagy” were especially enriched in P28 cortex (Figure 3D). Examination of the genes related to “ion transport” with Otx2-bound promoter showed that 21 out of 28 had P28-specific binding (Table 2, Figure S2E). Interestingly, part of “ion transporters” tends to be developmentally upregulated between P10 and P25 of fast-spiking interneurons in the somatosensory cortex (Okaty et al., 2009). In particular, potassium channels such as *Kcnc1* (K_V_3.1), *Kcna1/6* (K_V_1.1/1.6), and *Kcnk1* contribute to the maturation and fast-spiking properties of PV cells (Rudy and McBain, 2001; Okaty et al., 2009). Moreover, some glutamate and GABA receptor genes also had P28-specific Otx2 binding sites (Table S4). Notably, the two NMDA receptor subunit genes *Grin2a* and *Grin2b* possessed P28-specific and common Otx2 binding sites, respectively, consistent with the activity-dependent switch in predominant subunit composition from NR2B to NR2A during development (Williams et al., 1993; Flint et al., 1997). Thus, the list of genes that were bound by Otx2 specifically in postnatal (P28) cortex would indicate their functions to promote activity-dependent development of PV cell characteristics.

**Table 2:** Ion transporter genes with Otx2 binding sites and their developmental regulation.

**Gene**	**Otx2 binding site**	**Developmental regulation**
Hcn1^*^	P	N.A.
Kcna1^*^	P	Up
Kcna6^*^	P	Up
Kcnc1^*^	P	Up
Kcnc4^*^	P	N.A.
Kcnd2^*^	E/P	Down
Kcnd3^*^	P	N.A.
Kcnf1^*^	P	N.A.
Kcnh3^*^	P	N.A.
*Kcnh4*	P	N.A.
Kcnh7^*^	P	N.A.
Kcnip3^*^	E/P	N.A.
Kcnj2^*^	E/P	N.A.
*Kcnj4*	P	N.A.
*Kcnj9*	P	Up
Kcnk1^*^	P	Up
Kcnk4^*^	P	N.A.
Kcnk9^*^	P	N.A.
*Kcnn2*	E/P	Down
Kcnq3^*^	P	N.A.
Kcnq5^*^	P	N.A.
Kcns2^*^	P	Down
Nalcn^*^	P	N.A.
Slc9a8^*^	E/P	N.A.
Slc9a9^*^	E/P	N.A.
Tmem175^*^	P	N.A.
Tmem38a^*^	E/P	N.A.
*Scn8a*	P	Up

Genes carrying Otx2 binding sites within 5 kb around TSS in both embryo and P28 (E/P) or only in P28 cortex (P) are shown. Asterisks (^*^) mark genes involved in GO term “potassium ion transmembrane transport.” Data of developmental regulation are from Okaty et al. (2009). N.A., not available.

### Genes implicated in ocular dominance plasticity have Otx2 binding sites

Postnatal Otx2 induces the critical period of ocular dominance plasticity (ODP) in the V1 (Sugiyama et al., 2008). Therefore, we focused on genes implicated in ODP from mouse phenotypes and 32 candidate genes previously discovered by comparing three different data sets (components of ODP related pathways; genes regulated by monocular deprivation or dark-rearing; genes with correlation between ODP and expression levels; Rietman et al., 2012). As shown in Table 3, 28 out of the 54 known and candidate ODP genes inspected had Otx2 binding sites (full list of inspected genes is shown in Table S5). We noticed that some of the ODP genes with functional evidence such as *Gad2* (Hensch et al., 1998), *Grin2a* (Fagiolini et al., 2003), *Arc* (McCurry et al., 2010), *Clock* (Kobayashi et al., 2015), and *Csgalnact1* (Hou et al., in press) had strong Otx2 peaks at their promoters, suggesting that Otx2 may directly regulate these important critical-period plasticity genes. Of these, *Csgalnact1*, which encodes a key chondroitin sulfate (CS)-synthesizing enzyme, showed postnatal binding of Otx2 consistent with its role in increasing CSPG around developing PV cells (Hou et al., in press).

**Table 3:** Otx2 binding sites in ODP genes.

**ODP gene**	**Protein alias**	**Implicated**	**Sources**	**Otx2 BS**
*Clock*			Kobayashi et al., 2015	E/P
*Csgalnact1*	CS-T1	CSPGs	Hou et al., in press	P
*Otx2*			Sugiyama et al., 2008	E/P
*Ntrk2*	TrkB	BDNF	Huang et al., 1999	P
*Igfbp5*			Tropea et al., 2006	E/P
*Ddx6*			Tropea et al., 2006 candidate	P
*Arc*			Known^*^	P
*Gad2*	GAD65		Known^*^	E/P
*Grin2a*	NR2A		Known^*^	P
*Prkar2b*	PKA RII beta		Known^*^	E/P
*Rtn4r*	NgR		Known^*^	Genic
*Ache*		Acetylcholine	Candidate^*^	E/P
*Akap7*	AKAP15	PKA	Candidate^*^	P
*Camk2d*	CaMKII-delta	CamkII	Candidate^*^	P
*Cckbr*	CCK2R	ERK, GABA receptors	Candidate^*^	P
*Ctdsp2*	SCP2	Calcineurin	Candidate^*^	E/P
*Gabrg2*	GABA_A_R gamma2	GABA receptors	Candidate^*^	E/P
*Gnai1*	Gialpha1	Serotonin	Candidate^*^	E/P
*Mapk8*	JNK1	ERK	Candidate^*^	E/P
*Ncam1*		CSPGs, PSA	Candidate^*^	E/P
*Nlk*		ERK	Candidate^*^	E/P
*Npy5r*	Y5R	GABA receptors	Candidate^*^	P
*Phf21a*	BHC80	HDAC	Candidate^*^	E/P
*Phip*		IGF-1	Candidate^*^	E/P
*Ppm1l*	PP2C epsilon	Calcineurin	Candidate^*^	E/P
*Ppp1r1b*	PP1 sub. 1b	Calcineurin, BDNF	Candidate^*^	Genic
*Slc1a3*	GLAS	GABA receptors	Candidate^*^	E/P
*Zfp207*	Zep	CSPGs	Candidate^*^	E/P

ODP related genes carrying Otx2 binding sites within 5 kb around TSS in both embryo and P28 (E/P) or only in P28 cortex (P) are shown. “Genic” indicates that the Otx2 binding site in the gene is >5 kb distal from TSS. “Known^*^” and “Candidate^*^” are from Rietman et al. (2012) (see text). BS, binding sites.

In addition, comparison of ODP candidate genes with our ChIP-seq targets showed that 17 out of 32 (53%) of the candidate genes had Otx2 binding sites (Table 3). These genes contain factors implicated in GABA receptors and signaling pathways involving protein kinase A (PKA), extracellular signal regulated kinase (ERK), and calcineurin. In sum, our list of Otx2 target genes may provide additional criteria for the exploration of candidate genes for critical period plasticity.

### Dysregulated genes in Otx2-deficient interneurons

**Figure 4:**
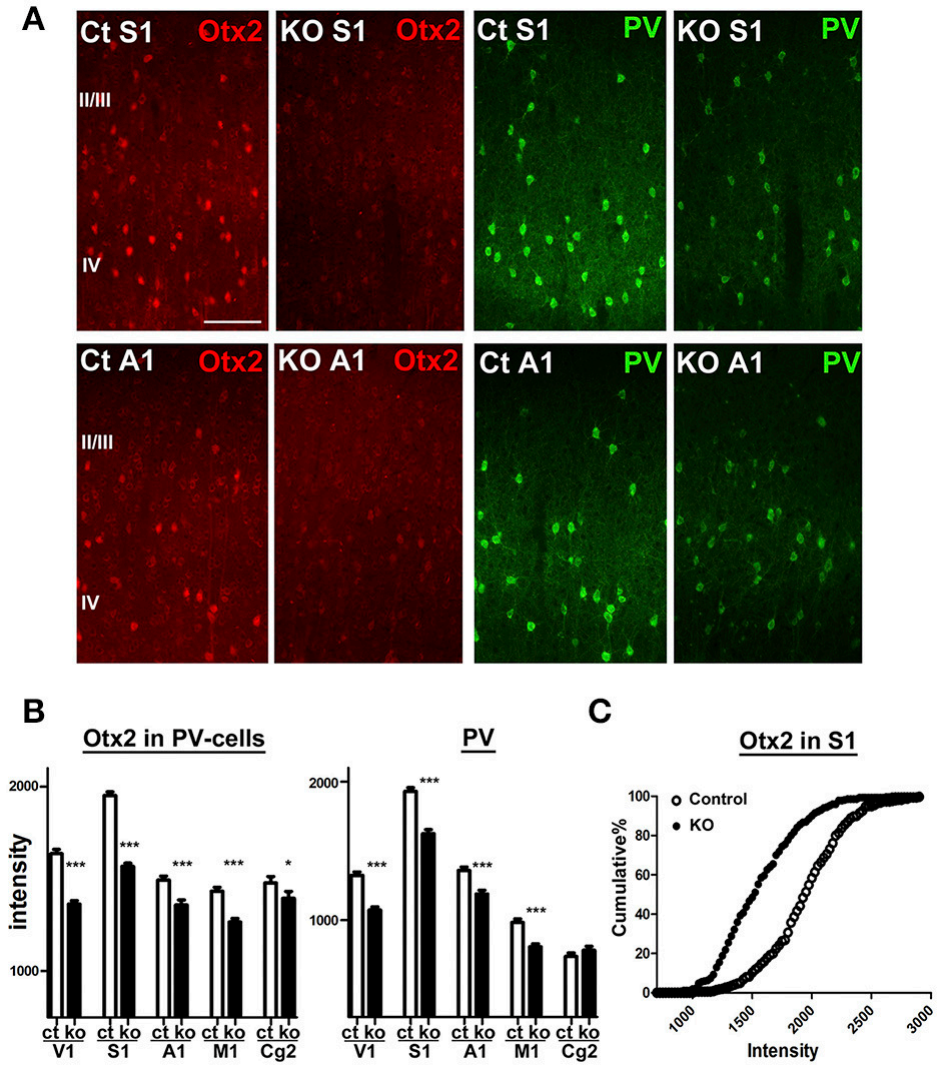
Otx2 reduction across cortical regions in conditional KO mice. **(A)** Reduction of Otx2 (red) and PV (green) immunoreactivities in the primary somatosensory (S1) and auditory (A1) areas in Otx2 KO mice at P28. The scale bar represents 100 μm. Ct, control. **(B,C)** Quantitative analysis of intensity for Otx2 and PV within PV-cell somata in supragranular layers of each cortical regions (Ct, control; M1, primary motor cortex; Cg2, cingulate area 2; control vs. KO, 171–339 cells for control, 178–321 cells for KO, *n* = 6, Mann–Whitney *U*-test, ^***^p < 0.0001; ^*^p < 0.05). Cumulative distribution reveals decreased intensity of Otx2 in the S1 of Otx2 KO mice **(C)**.

Next, we aimed to examine Otx2-dependent gene expression using the conditional KO mice in which Otx2 gene is deleted by Cre expression under control of *CamKII*α promoter. This conditional deletion of Otx2 in its expressing regions such as retina, thalamus and midbrain causes reduction of Otx2 protein in the V1 (Sugiyama et al., 2008), while cortical Otx2 is also derived from the choroid plexus (Spatazza et al., 2013). We quantified Otx2 immunoreactivity within PV cells of control (*Otx2*^*fl*/*fl*^) and Otx2-deficient (*Otx2*^*fl*/*fl*^; *CaMKII*α-*Cre*) mice on P28 and found that the fluorescence intensity of Otx2 was significantly reduced across the areas of Otx2-deficient cortex in addition to the V1 (Figure 4). PV protein was also reduced in these areas, suggesting Otx2 function in PV cell maturation. Thus, we used the KO mice (*Otx2*^*fl*/*fl*^; *CaMKII*α-*Cre*) to analyze Otx2-dependent transcriptome in cortical interneurons.

Interneurons tagged by Venus fluorescence under control of the VGAT promoter (*VGAT-Venus*; Wang et al., 2009) were sorted by flow cytometry from control and Otx2-deficient cortices and subjected to RNA-seq (Figure S3). We found that among all of the genes expressed in control interneurons, Otx2-bound genes were highly expressed in comparison to genes without Otx2 binding sites (Figure S4). To identify differentially expressed (DE) genes between the interneurons of control and Otx2-deficient cortices, we used a bioinformatics program (Cuffdiff) and ranked DE genes according to *q*-value (top 100 shown in Table S6). Interestingly, prominent DE genes were immediate early genes (*Fos, Fosb, Jun, Junb, Arc, Npas4*), which can be activated by cAMP or calcium ions in response to extracellular stimuli. Some of these activity-dependent genes (*Junb, Jund, Arc*) had P28-specific Otx2 binding sites, which suggests that Otx2 is involved in regulating these genes in postnatal interneurons. Regardless of Otx2 binding sites, dysregulation was seen for the genes related to amphetamine addiction (*Ppp1r1b, Adcy5, Arc, Fosb, Fos, Jun*) that are implicated in acceleration of dopamine transmission, and L-serine biosynthesis (*Phgdh, Psph, Psat1*) that provides the source for D-serine, a specific co-agonist of the NMDA receptor (Figure S5). Interestingly, these pathways are linked to schizophrenia (Bramness et al., 2012; Balu and Coyle, 2015), implying that Otx2 is involved in this disease.

**Figure 5:**
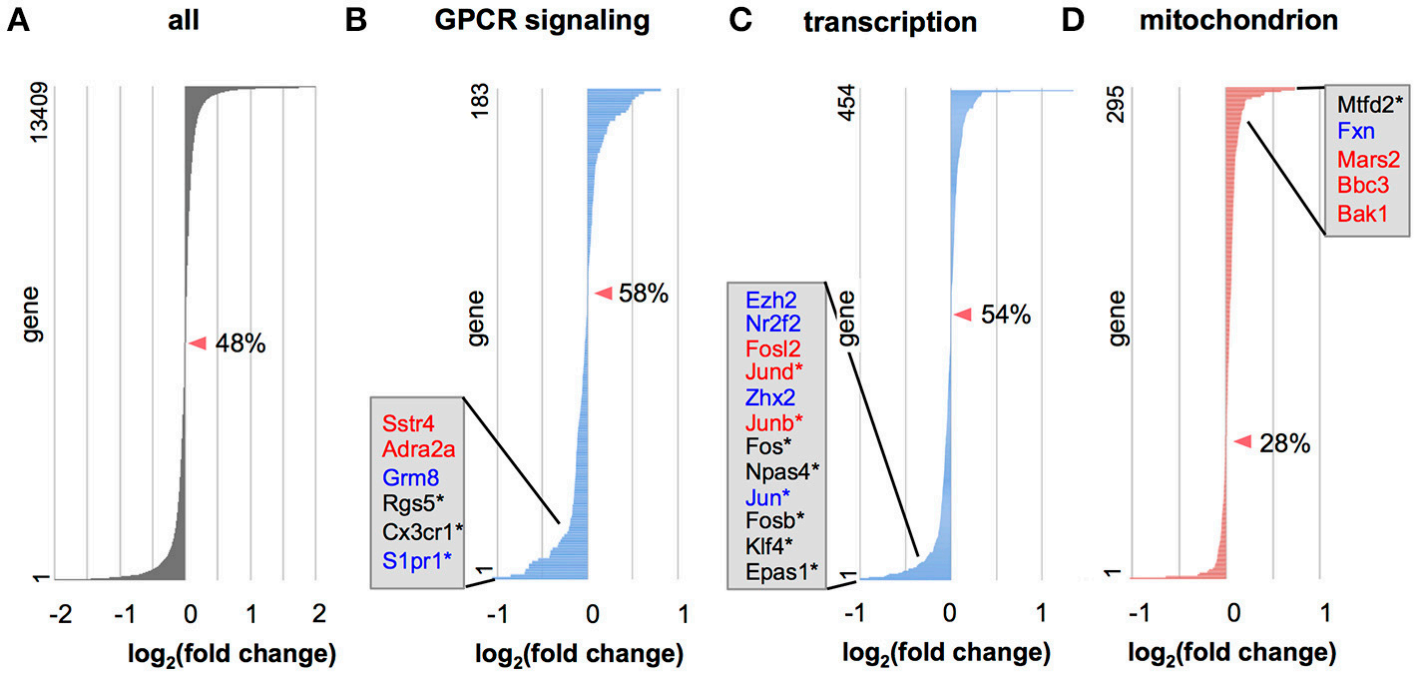
Gene categories dysregulated in Otx2-deficient interneurons. **(A–D)** Expression changes in Otx2-deficient interneurons in RNA-seq analysis for all genes **(A)** or in genes involved in the G protein-coupled receptor (GPCR) protein signaling pathway **(B)**, DNA dependent transcription **(C)** and mitochondria **(D)**. Genes are ordered according to their expression change compared to control interneurons. Zero-crossing points (no change) are indicated by arrowheads. Percentage (%) indicates ratio of decreased genes (fold change < 1) in the gene set. Representative DE genes with Otx2 binding sites (red: P28 only; blue: common to embryo and P28) or DE genes within top 100 as ranked in Table S6 (asterisks) are shown in each category.

To further examine whether genes in a defined set of particular functional category tend to be upregulated or downregulated, we applied gene set enrichment analysis (GSEA; Subramanian et al., 2005) to the data from all expressed genes. GSEA in biological processes revealed that several gene categories were significantly dysregulated (Tables S7, S8, Figure 5). Many gene categories enriched for Otx2-bound genes, such as “signal transduction,” “transcription,” “RNA processing,” “translation,” and “proteolysis” (Tables S2, S3), were dysregulated in Otx2-deficient interneurons (Tables S7, S8). Importantly, dysregulation of cellular respiration and metabolism indicated that mitochondrial function and homeostasis were impaired in Otx2-deficient interneurons. GSEA also revealed a striking dysregulation of the term “mitochondrion” in cellular components under Otx2-reduced conditions (Figure 5C), again suggesting an Otx2-dependent function in mitochondria. Together, these results suggest that a loss of Otx2 in cortical interneurons broadly affects the transcription of genes for several aspects of cellular functions in coordinating the development and maintenance of cortical interneurons.

### Otx2 regulates expression of the anti-oxidant gene *Oxr1* in cortical interneurons

**Figure 6:**
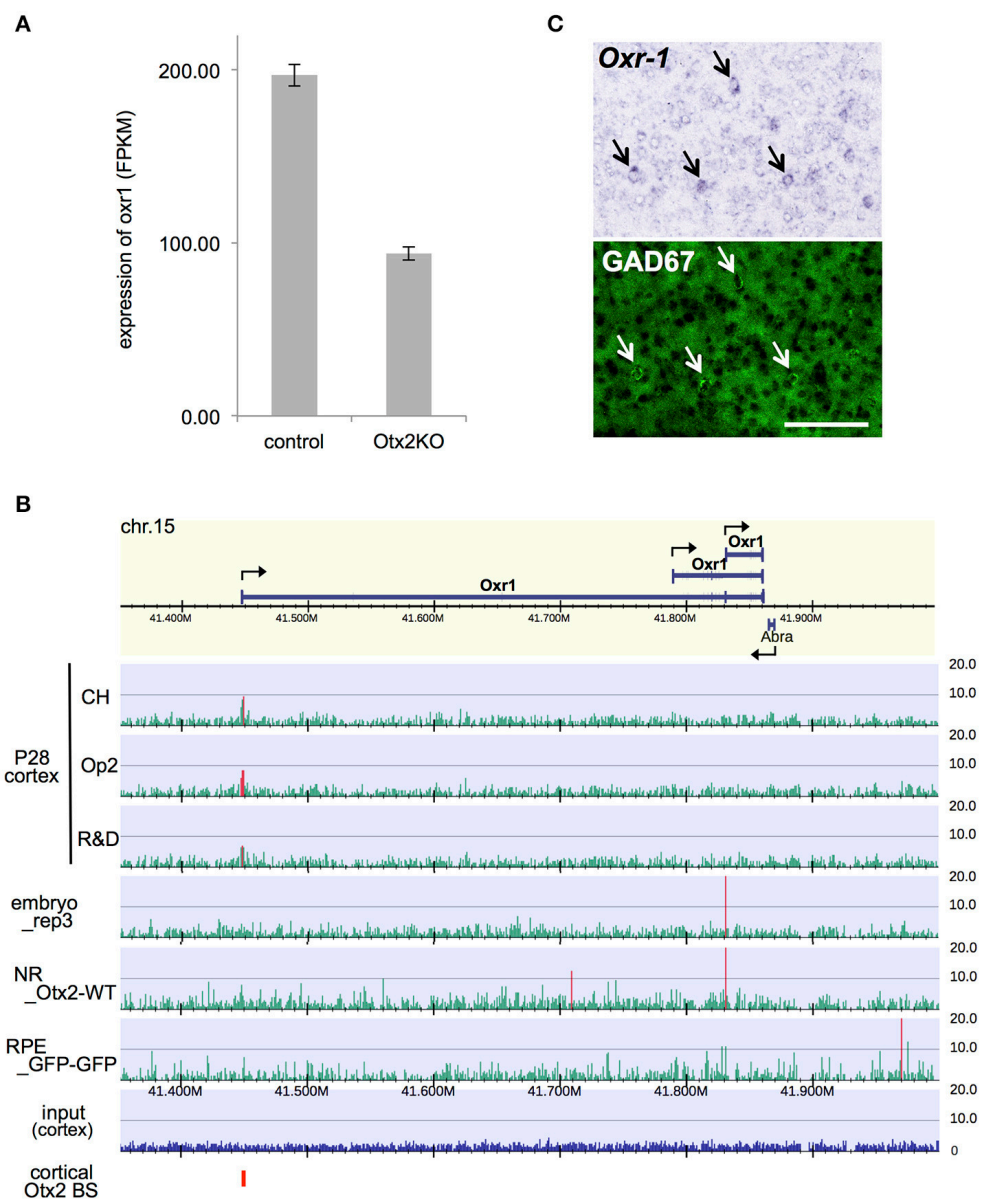
Otx2 regulates expression of anti-oxidant gene *Oxr1* in cortical interneurons. **(A)** Reduction of *Oxr1* expression in Otx2-deficient interneuron. Expression level is shown in FPKM (fragments per kilobase of transcript per million fragments). **(B)** Otx2 binding sites across *Oxr1* region in different stages/tissues. NR, neural retina. **(C)** Co-localization of *Oxr1* transcripts (*in situ* hybridization) and GAD67 immunofluorescent signal (green) in the primary visual cortex of P28 mouse. Arrows indicate *Oxr1*- and GAD67-positive interneurons. Scale bar, 100 μm.

Among the DE genes in our RNA-seq analysis, a notable gene whose expression was significantly downregulated in Otx2-deficient interneurons was *oxidative resistance-1* (*Oxr1*; Figure 6A). Oxr1 is reported to play an important protective role against oxidative stress-induced neurodegeneration *in vitro* and in the cerebellum (Oliver et al., 2011), and in regulating mitochondrial morphology (Wu et al., 2016a). Otx2 bound to the promoter region of *Oxr1*, indicating direct transcriptional regulation (Figure 6B). Interestingly, the Otx2 ChIP-seq peak at the promoter of full-length *Oxr1* was prominent only in P28 cortex, whereas the TSS of a shorter isoform was marked in embryonic subpallium and neural retina (Figure 6B, Figure S6), suggesting that expression of *Oxr1* transcription isoforms might be regulated differently in P28 cortex. Both full length (Oxr1-FL) and short isoform peptides (Oxr1-C) contain the conserved carboxyl-terminal domain, which is essential and sufficient for anti-oxidative function, but the presence of the extended amino-terminal region in Oxr1-FL confers additional regulatory properties (Finelli et al., 2016). Although Oxr1 is reportedly expressed in postnatal cortex (Allen Mouse Brain Atlas, http://mouse.brain-map.org), cell type specificity is unknown. We performed *in situ* hybridization and revealed that *Oxr1* transcript was enriched in GABAergic neurons in the visual cortex of P28 mice (Figure 6C), pointing to the importance of this gene in interneuron-specific function. Together, our data suggests that Otx2 may contribute to the protection of PV cells against oxidative stress-induced damage through transcriptional regulation of *Oxr1*.

## Discussion

In this study, Otx2 targets in juvenile cortical interneurons were investigated using ChIP-seq and interneuron-specific RNA-seq analysis. Our results demonstrate the existence of postnatal Otx2 binding sites around gene promoters and suggest that Otx2 modulates the expression of Otx2-bound genes related to the postnatal development and functional integrity of PV interneurons.

Our ChIP-seq analyses revealed that more than half of the Otx2 binding sites were within 5 kb of the TSS and only 7% were assigned to intergenic regions in the P28 cortex genome (Figure 1). Interestingly, binding sites in the promoter region showed greater enrichment postnatally than at all other ages and in all other cell types. For example, the distribution of Otx2 binding sites is enriched mainly in the distal part of genes in embryonic subpallium and postnatal RPE (Samuel et al., 2014; Hoch et al., 2015). Even in the neural retina, which has more TSS-centered binding sites than RPE, 26% of the binding sites are in the distal intergenic position (Samuel et al., 2014). Accordingly, our examination of the overlap of Otx2 binding sites showed a small number of common sites among these different sources. Thus, our results emphasize that Otx2 binds to distinct genomic sites depending on cellular context.

*In vivo* binding sites of transcription factors would be determined by combinations of several factors such as presence of consensus sequence, interactions with specific partner transcription factors, and cell-type specific chromatin structure including histone modifications and DNA methylation status. Notably, most of the Otx2 binding sites in P28 cortex overlapped with cell-type specific open chromatin sites in adult cortical neurons (Mo et al., 2015, data not shown), indicative of functional cis-regulatory sites. Further analysis of epigenetic marks in juvenile PV cells would provide insight into Otx2 binding sites with relevance to chromatin states and functional expression.

Consistent with Otx2 localization in cortical PV cells (Figures 1, 4, Sugiyama et al., 2008; Spatazza et al., 2013), Otx2-bound genes such as *Gad1/2* (GAD67/65) and *Ntrk2* (TrkB receptor) were especially related to neuronal development and functional maturation of cortical interneurons (Figure 2). Otx2-bound genes that appear only in P28 cortex but not in embryo are prominently involved in vesicle-mediated and ion transport (Figure 3, Table 2). Of these, potassium ion transporters such as the K_V_3.1 family are known to be important for fast-spiking activity of mature PV cells (Rudy and McBain, 2001). Therefore, Otx2 may directly modulate gene expression for functional maturation of PV interneurons, and hence for critical period plasticity (Table 3).

GSEA following RNA-seq analyses of cortical interneurons revealed that several gene sets were significantly dysregulated in Otx2-deficient interneurons (Figure 5, Tables S7, S8). A number of GO categories were actually enriched in Otx2-bound genes (Figures 2, 3, Tables S2, S3), indicating that Otx2 directly regulated these to cause expression changes under conditions of its insufficiency. However, in contrast to thousands of Otx2 ChIP-seq peaks, our interneuron-specific RNA-seq identified only a small number of genes whose expression level differed significantly between control and Otx2 KO cells (Table S6). Moreover, fold-change of expression level in Otx2 KO cells was rather moderate compared to that in other models like Clock KO PV cells (Kobayashi et al., 2015). In ChIP-seq analysis, generally, one cannot rule out the possibility that data include non-specific targets and miss critical targets. Our Otx2 ChIP-seq peaks were considered to be sufficiently reliable given that more than two distinct antibodies could recognize the sites while weak but important peaks could be recovered by using lower threshold for peak calling. Thus, we speculate two reasons for the small number of apparent DE genes despite the relatively large number of Otx2-bound genes.

First, Otx2 binding sites are actually functional, but the expression levels of the bound genes may not alter until the cells receive specific stimuli/signals. In this case, Otx2 may render the chromatin environment around its binding sites capable of an immediate response to additional signals such as the binding of other transcription factors or modification of regulatory factors. Interestingly, Otx2 is required for such a “poised” state of histone modifications of target promoters in medulloblastoma cells (Bunt et al., 2013). Otx2-bound genes such as *Arc, Ddx6*, and *Igfbp5*, whose expression changes by neuronal activity or by monocular deprivation during the critical periods in visual cortex (Majdan and Shatz, 2006; Tropea et al., 2006), would be good candidates for investigation of Otx2-dependent expression. Second, although we focused on interneurons as the source of RNA-seq, cell populations are still heterogeneous: (1) The samples contained different cortical regions in which thousands of genes are differentially expressed, although the differences between developmental stages are much more prominent (Benoit et al., 2015). If the timing of cell maturation differs among regions, gene expression would be even more diverse, leading to considerable dispersion. (2) Interneurons themselves are diverse in specific cell types (Kawaguchi and Kubota, 1997). Not all interneurons possess Otx2, making it difficult to clarify Otx2-dependent gene expression in our system. Nevertheless, Otx2-bound genes showed a higher expression than non-bound genes (Figure S3). Otx2 may modify the genes that have apparent expression and thus, presumably, an important function in interneurons.

Our results from ChIP-seq and RNA-seq analyses suggest that Otx2 regulates various functional categories. The most enriched categories were transcription and chromatin modifications, indicating the function of Otx2 as a master regulator of transcriptional networks. Moreover, genes for RNA processing (splicing) and translation were markedly enriched. Considering the function of homeoproteins in protein translation as shown for Engrailed (Brunet et al., 2005), Otx2 may regulate each process of protein expression from transcription to translation.

Our data also indicated that Otx2 targets transmits intracellular signaling in response to external signals or neuronal activity, thus promoting neuronal maturation and rewiring neural networks. The expression of Otx2-bound genes related to the G protein/small GTPase-mediated signaling pathway was impaired in Otx2-deficient interneurons (Figure 5). In fact, the mutant interneurons seemed to have an immature expression profile, with upregulation of genes involved in metabolism (Table S8). Augmentation in metabolisms is similarly observed in immature visual cortex in dark-reared mice, considered as a result of homeostatic scaling to enhance overall activity (Tropea et al., 2006).

Interestingly, Otx2 targets included genes related to mitochondrial components and oxidative stress. Otx2-bound genes involved in autophagy/mitophagy were enriched only in P28 cortex (Figure 3), indicating postnatal regulation of mitochondrial damage and turnover by Otx2. Notably, expression of genes in mitochondria-related terms including “Alzheimer's disease” and “oxidative phosphorylation” are reported to be enriched in juvenile cortex (Benoit et al., 2015) and were all dysregulated in Otx2-deficient interneurons (Figure 5). During development, PV cells acquire their characteristic ability to generate fast-spiking action potentials, which demands high energy mitochondrial metabolism and response to oxidative stress as a result (Do et al., 2015). Therefore, one of the important aspects of postnatal Otx2 may be maintenance of mitochondrial homeostasis; in other words, a balance between metabolism and stress in the developing PV cell. Indeed, the function of Otx2 in cellular homeostasis has been demonstrated in mature retina (Housset et al., 2013), where Otx2 ensures mitochondrial function by modulating its components (Kim et al., 2015). As a direct target of Otx2 in the cortex, Oxr1 would be a key mediator of cellular integrity, considering its roles in antioxidant defense systems and maintenance of mitochondrial morphology (Oliver et al., 2011; Wu et al., 2016a). Because the protective function of Oxr1 is shown for cerebellar neurons in mouse (Oliver et al., 2011), future study may elucidate the role of Oxr1 in cortical interneurons.

Regulation of redox homeostasis is of great importance in fast-spiking PV cells and PNN formation around them, which are implicated in the critical period of ODP (Morishita et al., 2015a,b) and schizophrenia (Cabungcal et al., 2013a). The PNN protects PV cells from oxidative stress; conversely, PNN itself is vulnerable to oxidative stress, especially in juvenile brain (Cabungcal et al., 2013a,b). In fact, Otx2 promotes PNN formation perhaps by directly upregulating the expression of *Csgalnact1* (Table 3) and PNN components (Sugiyama et al., 2008; Hou et al., in press), indicating a requirement for Otx2 in protecting PV cells from oxidative stress. Interestingly, the circadian factor CLOCK, which also plays a role in PV cell maturation and ODP in the visual cortex, regulates downstream genes involved in respiratory chain and redox regulation (Kobayashi et al., 2015). Remarkably, we found that several genes for circadian rhythms including *Clock* were enriched among Otx2-bound genes. Thus, Otx2 may guarantee functional homeostasis of PV cells by dealing with metabolism and oxidative stress through direct and indirect regulation of various factors including PNN and circadian genes.

Accumulating evidence strongly indicates a functional deficit of PV cells in developmental and neuropsychiatric disorders such as schizophrenia and autism spectrum disorder. Oxidative stress in PV cells arising from aberrant mitochondrial/metabolic regulation is one of the hallmarks of these disorders (Do et al., 2015). Consistent with this, *Otx2* mutations have been reported in people with such developmental disorders (Schilter et al., 2011) and polymorphism in *Otx2* is a risk factor for bipolar disorder, whose etiology is also related to mitochondrial dysfunction (Sabunciyan et al., 2007). Therefore, our results in Otx2 targets suggest plausible candidates for therapeutic strategies in maintaining and recovering physiological homeostasis in PV cells.

## Materials and methods

### Animals

All animal experiments were conducted in compliance with a protocol approved by the Institutional Animal Care and Use Committee of Niigata University (Permit Number: #27-SHINDAIKENDAI39-3). Conventionally raised C57BL/6J mice (12:12 h light:dark cycle) were purchased from Japan SLC. The *Otx2*^*fl*/*fl*^ mice (Tian et al., 2002) were crossed with *CaMKII*α-*Cre* mice (Minichiello et al., 1999) to generate mice with a conditional Otx2 KO in postnatal brain (Sugiyama et al., 2008). To tag interneurons for FACS, *Otx2*^*fl*/*fl*^; *CaMKII*α-*Cre* mice were further crossed with *VGAT-Venus* mice (Wang et al., 2009).

### Chromatin immunoprecipitation (ChIP) and quantitative real-time PCR (qPCR)

A large part of the cerebral cortex (from Bregma +1 mm to posterior end) was dissected from P28 C57BL/6J mice and flash-frozen in liquid nitrogen. Pooled cortices from 10 males and 10 females (total 20 mice) were cross-linked with 1% formaldehyde in PBS for 15 min at room temperature and ChIP was performed using the ChIP-IT High Sensitivity kit (Active Motif) per the manufacturer's instructions, with some modifications: after homogenization using a Dounce homogenizer, nuclei were washed in LB0 buffer (20 mM Tris-HCl [pH7.5], 10 mM NaCl, 1 mM EDTA, 0.2% NP-40, 1 mM PMSF) and resuspended in MNase buffer (10 mM Tris-HCl, pH7.5, 10 mM NaCl, 2.5 mM MgCl_2_, 0.1% NP-40, 1 mM DTT, 1 mM PMSF), provided with cOmplete™ EDTA-free protease inhibitor cocktail (Roche Diagnostics). Sonication was carried out using a Misonix 2000 Sonicator (Misonix) at power 7. After four pulses of 25 s each, 5 mM CaCl_2_ and MNase (NEB, 3,000 U in 500 μl reaction mixture) were added and incubated for 10 min at 37°C. The reaction was stopped by adding 20 mM EGTA. The samples were diluted by half with 2 × ChIP dilution buffer (2% Triton X-100, 280 mM NaCl, 80 mM Tris-HCl [pH8.0] in ChIP buffer from the Active Motif kit) and further sonicated for two pulses of 25 s each. Insoluble material was removed by centrifugation at 15,000 rpm for 5 min at 4°C. Fragmented chromatin sample was divided into four aliquots, of which three were immunoprecipitated with three independent anti-Otx2 antibodies: CH (Millipore AB9566, 4 μl polyclonal rabbit serum), R&D (R&D systems AF1979, 4 μg polyclonal goat IgG) and Operon2 (generated in this study, 6 μg polyclonal rabbit IgG raised against full-length Otx2). Normal rabbit IgG (Abcam ab46540) and normal goat IgG (Santa Cruz sc-2028) were used as negative controls. For immunoprecipitation with CH antibody only, which is not affinity-purified, chromatin was pre-cleared by incubating with protein G agarose-bound normal rabbit IgG for 1 h to increase the signal-to-noise ratio. Samples were assessed for enrichment by qPCR with SsoAdvanced Universal SYBR Green SuperMix (BioRad). Primers are listed in Table S9.

### ChIP-Seq

DNA from whole cell extracts (input) and ChIP fractions was further sheared to an average size of ~150 bp by ultrasonication (Covaris), end-repaired, ligated to sequencing adapters and amplified using the NEBNext ChIP-seq Library kit (New England Biolabs) according to the manufacturer's instructions. The DNA library was sequenced on the Illumina HiSeq2000 platform to generate single-end 65-bp reads. Sequenced reads of both ChIP fractions and input were aligned to the mouse genome (UCSC mm10) using Bowtie version 1.1.0 (Langmead et al., 2009), allowing two mismatches in the first 28 bases per read (-n2 option). All duplicate reads and those without unique alignment were removed from further analysis. In average, total of 46,645,691 reads (80.1%) were mapped uniquely (Table S10). Peak calling was performed using MACS software (Zhang et al., 2008; Feng et al., 2012) in version 2.1.1, using the nomodel option at a *q*-value threshold of <0.1. For visualization of peaks on the genomic map, DROMPA2 tool (Nakato et al., 2013) was used. Peaks that overlapped among different samples were calculated using BEDTools (Quinlan and Hall, 2010) and DROMPA2 (Nakato et al., 2013). As for defining the consensus set of Otx2 peaks from ChIP-seq using three different antibodies, each peak detected by MACS was first extended to 1,000 bp and then overlaps were calculated. Overlapped peak regions <500 bp were then extended to 500 bp. As one peak from a dataset can overlap with two peaks from another dataset, total peak number may vary in some cases. For comparison with published Otx2 ChIP-seq studies, fastq files of the following data from the database were used to perform mapping and peak calling in the same way as our data: GSE69724 for embryonic subpallium (Hoch et al., 2015) and GSE54084 for neural retina and RPE (Samuel et al., 2014). Number of reads and mapped ratio of each sample are summarized in Table S10. To compare the peak position to genes and TSS, DROMPA2 (Nakato et al., 2013; Figures 1B, 3B) and Genomic Regions Enrichment of Annotations Tool (GREAT; McLean et al., 2010; Figure 1C) were used, respectively.

### Isolation of interneurons

Interneurons were isolated using a trehalose-supplemented protocol (Saxena et al., 2012) from *Otx2*^*fl*/*fl*^*; VGAT-Venus*, or *Otx2*^*fl*/*fl*^*; CamKII*α*-cre; VGAT-Venus* juvenile mice (P27–30). In brief, digestion and dissociation of the same region of cerebral cortex as described in the ChIP procedure were carried out using the Papain Dissociation System (Worthington Biochemical Corporation) in the presence of 10% (vol/vol) D-trehalose (Wako Pure Chemical Industries). Digested tissue was dissociated with trituration to obtain a single-cell suspension. Hoechst 33342 (5 μg/ml) and propidium iodide (PI; 1 μg/ml) were added to the medium to identify viable cells. Using FACSAriaII (BD Biosciences), the Venus-fluorescent, PI-negative interneurons were sorted directly into Trizol LS reagent (Ambion) and flash-frozen in liquid nitrogen until RNA purification.

### RNA-Seq

RNA isolations were performed using the NucleoSpin RNA kit (Macherey-Nagel) or RNeasy plus universal MiniElute Kit (Qiagen). Eluted RNA was concentrated with NucleoSpin RNA Clean-up XS (Macherey-Nagel). RNA quality was assessed using the Agilent 2100 Bioanalyzer with the Nano LabChip Kit (Agilent Technologies). RNA of interneurons from one or two mice was mixed to obtain ~100 ng of total RNA for construction of one library. The mouse Truseq Stranded Total RNA with riboZERO kit (Illumina) was used to construct four libraries, which were then sequenced on the Illumina HiSeq2000 platform to generate single-end 65-bp reads. The resulting reads were aligned to the mouse genome (UCSC mm10) using TopHat v2.1.0 (Trapnell et al., 2009, 2012; Kim et al., 2013). The number of reads and mapped ratio of each sample, and the sex of the animals used, are summarized in Table S10. To identify differentially expressed genes (DEGs), we used Cuffdiff v.2.2.1 (Trapnell et al., 2009, 2012) to compare control and Otx2 KO samples, each of which had four biological replicates. Expression of each transcript was quantified as fragments per kilobase of transcript per million fragments mapped (FPKM) by Cufflinks v.2.2.1 (Trapnell et al., 2009, 2012). To calculate differences in gene expression among Otx2 bound- and unbound- genes, those with FPKM > 0.3 were considered as expressed and the ANOVA *p*-value was computed using the statistical toolkit R.

### Functional annotation and GSEA

GO and KEGG pathway functional annotations were performed using the NIH web based tool DAVID v.6.8 (Huang et al., 2009a,b). The web tool FunDo (Osborne et al., 2009) was used for annotation to Disease Ontology. GSEA (Subramanian et al., 2005) was used to identify functionally related groups of genes enriched toward the top or bottom of a ranked list. Genes were ranked in order of log_2_ (fold change) calculated by Cuffdiff, and genes with a status of “NOTEST” excluded from analysis. Gene names were converted from mouse to human, and the pre-ranked list was submitted to GSEA using the default parameters and the Molecular Signature Database (MSigDB; Subramanian et al., 2005) as the gene sets database.

### Immunohistochemistry

For immunohistochemistry, deeply anesthetized mice were perfused transcardially and post- fixed with 4% paraformaldehyde (Sugiyama et al., 2008). Floating coronal sections (20 μm) were incubated with the following primary antibodies: rabbit anti-Otx2 (Millipore AB9566), mouse anti-PV (Swant PV235), mouse anti-GAD67 (Millipore MAB5406). Alexa-conjugated anti-mouse and anti-rabbit IgG (Invitrogen) were used for secondary detection.

Quantitative analysis of fluorescence intensity was performed as described previously (Sugiyama et al., 2008). Immunostaining for experimental control and sample sections was performed concurrently with the same solutions, and images were photographed in one sitting with the same gain and exposure time. The fluorescence intensity for each cell was measured with the spot module of NIS-Elements AR Analysis software (Nikon) in the supragranular layers (a 600 × 350 μm area for V1 or A1, a 600 × 400 μm area for M1, a 600 × 500 μm area for S1 or Cg2). Otx2+/PV+ cells were defined by combining threshold (between the intensity values of 306–4,096) and area size (above 90 μm^2^) to distinguish positive cells from background signal. For quantification, we compared the data between two groups (Mann–Whitney *U*-test).

### *In situ* hybridization

*In situ* hybridization on sections mounted on glass slides was performed according to methods described previously (Sugiyama et al., 2008). In brief, coronal sections (20 μm) were hybridized at 65°C with a Digoxigenin (DIG)-labeled antisense RNA probe for *Oxr1*, then detected by alkaline phosphatase-conjugated anti-DIG antibody. For immunostaining of Gad67, sections were incubated with mouse anti-Gad67 antibody (Millipore MAB5406), and Alexa-conjugated anti-mouse IgG (Invitrogen) was used as a secondary antibody.

## Accession code

Sequencing data have been deposited at NCBI Sequence Read Archive (SRA) under accession number SRP099973.

## Author contributions

AS and SS designed the study. AS performed all experiments with help by NH and TI. AS, RN, YL, and XH performed bioinformatics analysis. AS, RN, XH, RK, SO, KS, and SS discussed the data. YY produced VGAT-Venus mice. AS and SS wrote the manuscript with input from other authors.

### Conflict of interest statement

The authors declare that the research was conducted in the absence of any commercial or financial relationships that could be construed as a potential conflict of interest.
