# Supplementary material for: Genome-Wide Target Analyses of Otx2 Homeoprotein in Postnatal Cortex
Source: Front Neurosci. 2017 May 31;11:307. doi: 10.3389/fnins.2017.00307 (PMC5450002; doi:10.3389/fnins.2017.00307)
Supplement: Supplementary file 2 [file Image1.PDF]

Sakai Figure S1

A

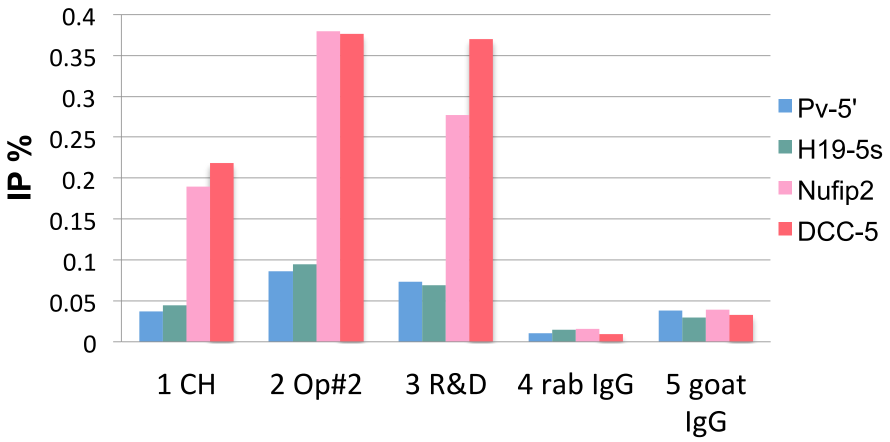

B

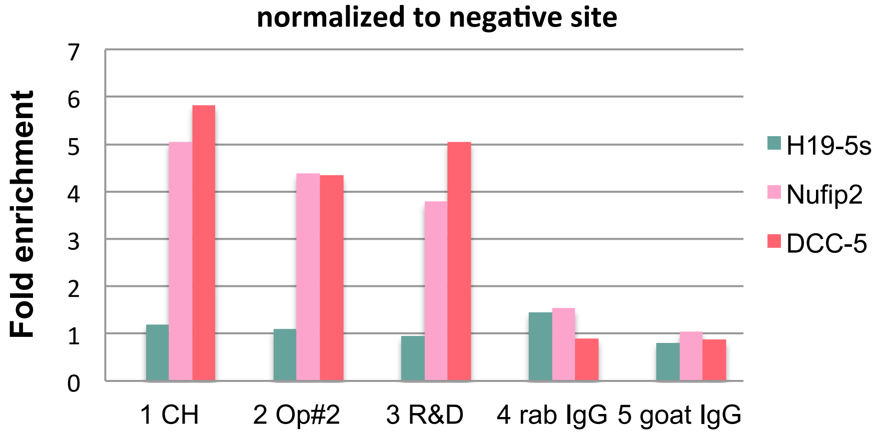

Figure S1. ChIP-qPCR confirmation of Otx2 binding sites.
(A) Compared with control IgGs, the three antibodies against Otx2 gave enrichment of the positive sites (Nufip2, DCC-5). **(B)** Fold enrichment relative to a negative site (Pv-5'). Compared to another negative site (H19-5s), positive sites were enriched ~6-fold only by Otx2-specific antibodies.

Sakai Figure S2

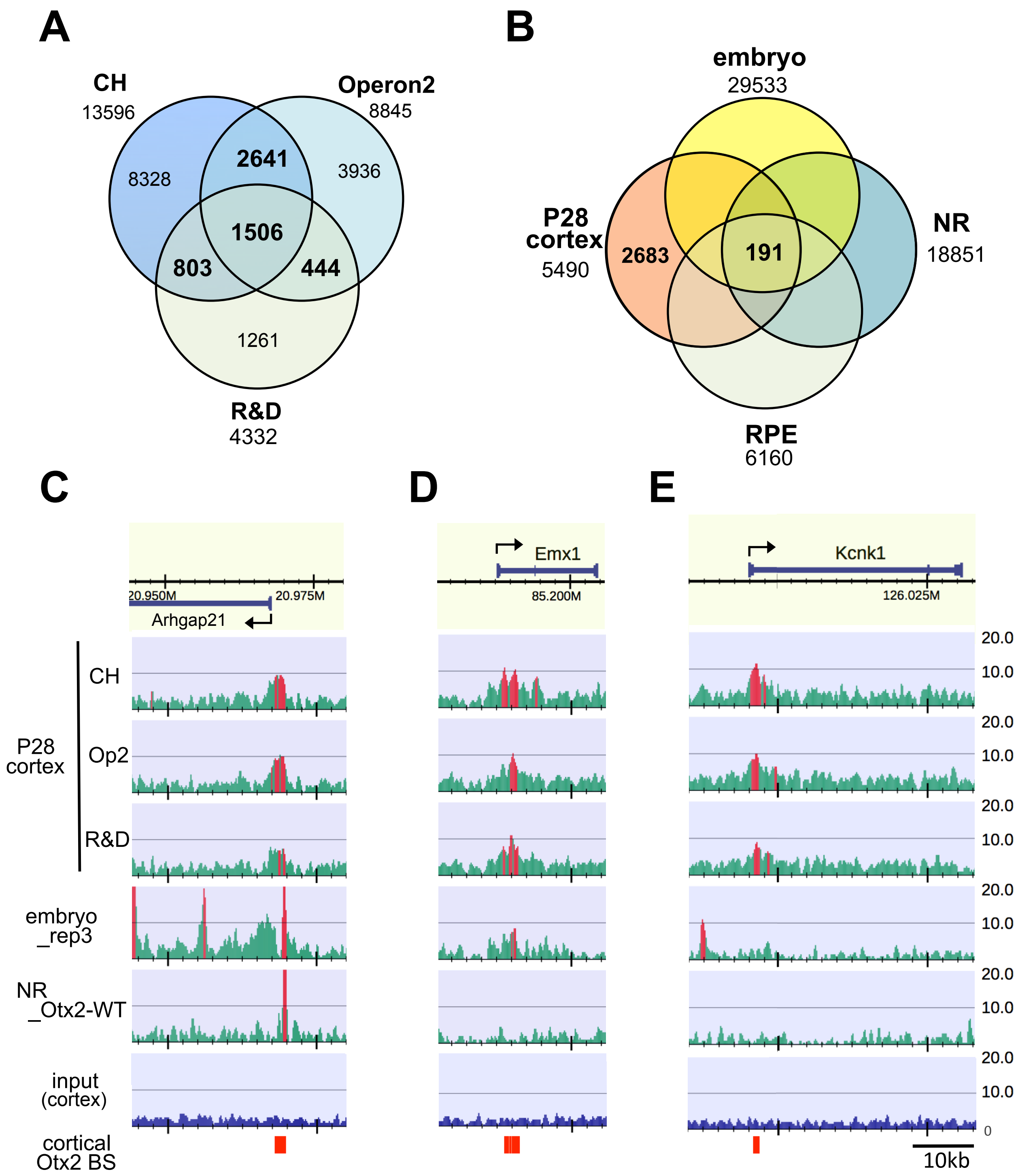

Figure S2. Overlapping ChIP-seq peak analysis.
(A) Venn diagram showing overlap of Otx2 peaks using three different antibodies for ChIP-seq from P28 cortex. Peaks that are common to two or three different antibodies (indicated in bold numbers) were considered as a consensus set of Otx2 binding sites in P28 cortex and used for further analyses. Note that the total number of common peaks is 5,490, which differs from simply adding overlapping peak numbers because of the possible overlap of one peak with two from another sample. **(B)** Venn diagram showing overlap of Otx2 peaks in different stages/cell types. ChIP-seq data sets of P28 cortex, subpallium from embryonic forebrain (embryo), mature neural retina (NR) and retinal pigment epithelium (RPE) are compared. **(C–E)** Examples of mapping patterns of Otx2 common among different sources **(C)**, common to P28 and embryo **(D)** and specific to P28 cortex **(E)**. Peaks and direction of transcription are shown as described in **Figure 1B**.

Sakai Figure S3

A

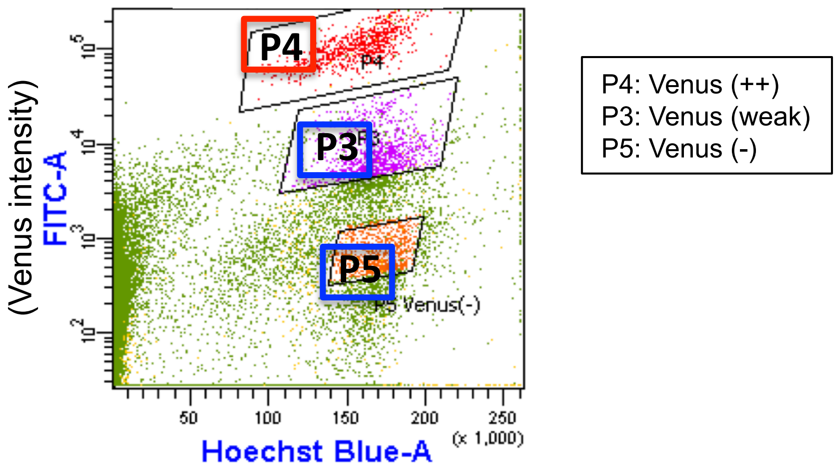

B

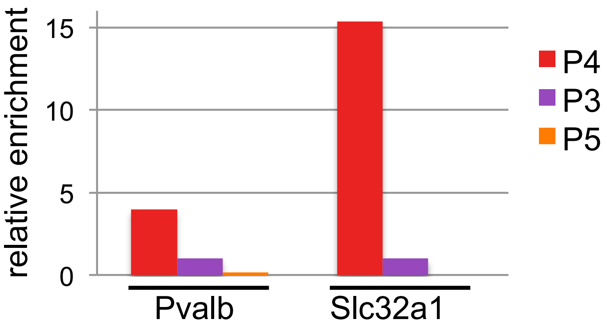

Figure S3. Sorting of interneuron.
(A) Cell population with strong Venus signal (P4; red cells) was collected as interneuron specific-fraction. **(B)** Verification of enrichment of interneuron. cDNA synthesized from each population was assayed for expression level of *Pvalb* (PV) or *Slc32a1* (VGAT) mRNA by RT-qPCR. Enrichment in the P4 fraction was calculated by comparison to the P3 (weak Venus signal) fraction.

Sakai Figure S4

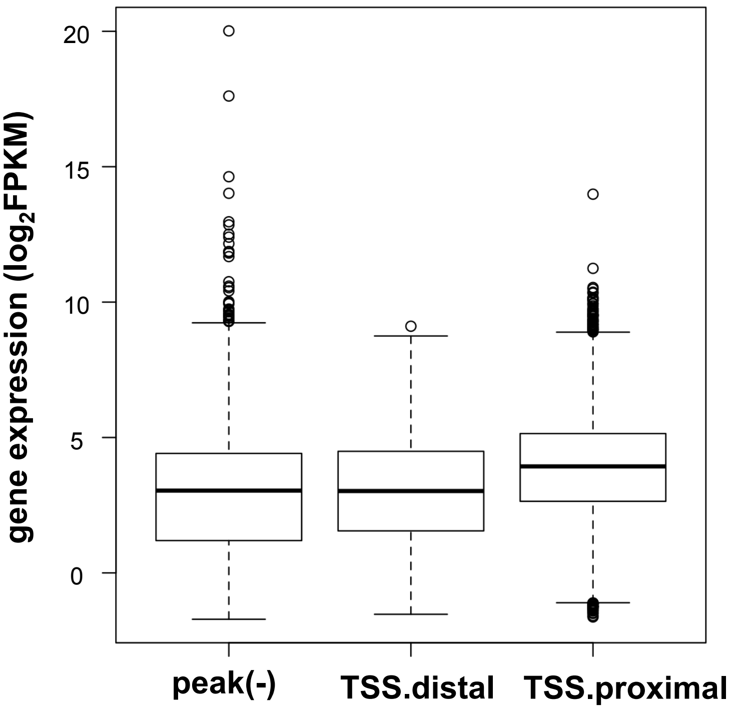

Figure S4. Gene expression in relation to Otx2 binding sites.
Expression scores (FPKM > 0.3) for genes with Otx2 binding sites within ± 5 kb of TSS (TSS.proximal) display significantly higher expression than those without binding sites (peak(−)) or those with binding sites > 5 kb away from TSS (TSS.distal) ($P < 2.2E{-}16$; one-way ANOVA).

Sakai Figure S5

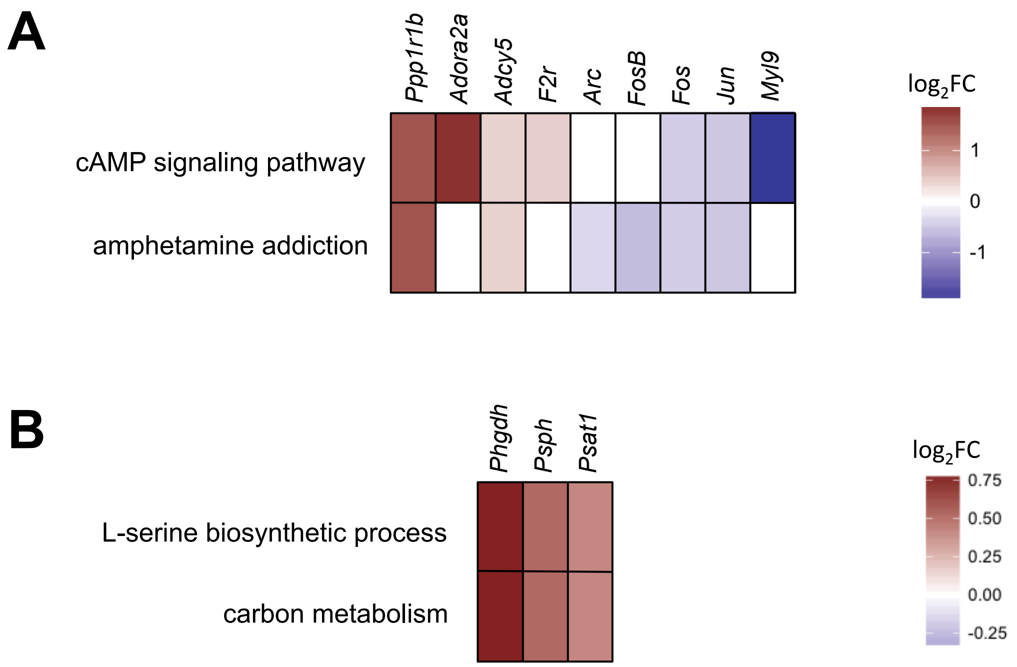

Figure S5. Clustering analysis of differentially expressed genes in Otx2-deficient interneurons.
(A, B) Heatmap shows $\log_2$-fold change (FC) of genes annotated with given GO term or KEGG pathways.

Sakai Figure S6

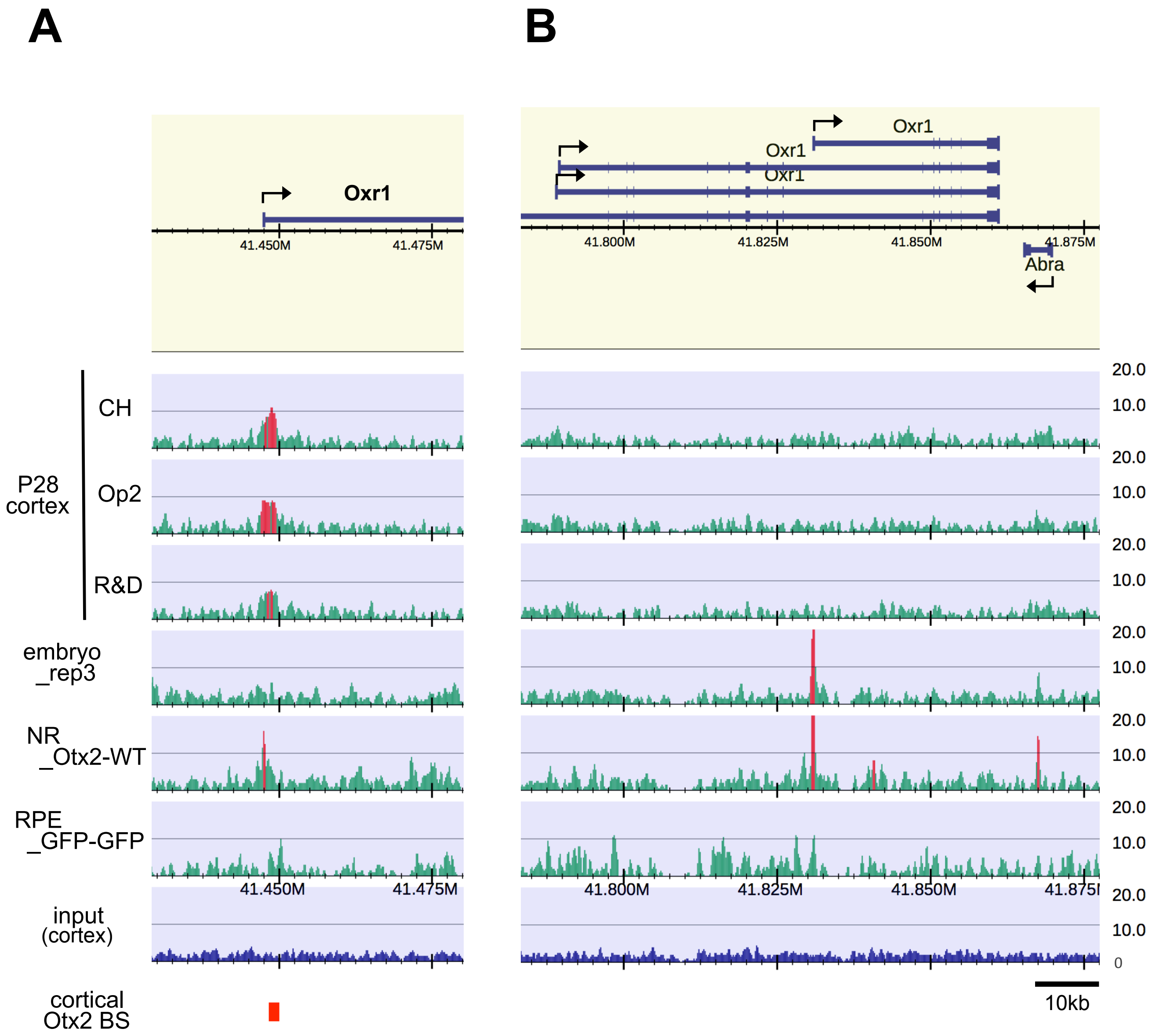

Figure S6. Magnified view of Otx2 ChIP-seq map around *Oxr1* region.
(A,B) Otx2 binding sites at *Oxr1* region around TSS of longest isoform **(A)** or at common 3' region including shorter isoforms **(B)**. Peaks and direction of transcription are shown as described in **Figure 1B**.
